# Knowledge, attitude, and practice toward pediatric vitamin D deficiency among parents

**DOI:** 10.3389/fped.2024.1393488

**Published:** 2024-06-28

**Authors:** Chunli Yu, Jingjuan Cai, Chunxiang Wang, Yan Luo, Jian Fang

**Affiliations:** Pediatric Medicine Department, Chengdu Children’s Specialized Hospital, Chengdu, China

**Keywords:** knowledge, attitude, practice, vitamin D deficiency, parents, children, cross-sectional study

## Abstract

**Objective:**

To investigate the knowledge, attitude, and practice (KAP) towards pediatric vitamin D deficiency (VitD) among parents and explore the risk factors of their knowledge, attitude, and practice.

**Methods:**

This cross-sectional study enrolled parents in our Hospital between November 2022 and January 2023.

**Results:**

A total of 621 valid questionnaires were collected in this study. The knowledge, attitude, and practice scores were 6.13 ± 3.07 (theoretical score range: 0–13), 31.13 ± 6.20 (theoretical score range: 9–45), and 27.47 ± 4.21 (theoretical score range: 9–45), respectively; the mean knowledge score was <60%, indicating poor knowledge. Commercial and service industry workers and a monthly income ≥5,000 CNY were independently associated with sufficient knowledge (all *P* < 0.05). The knowledge score, ethnic minorities, divorced/widows, and spouses with a master's degree or above were independently associated with positive attitudes (all *P* < 0.05). The attitude score, female, non-urban, undergraduate education, commercial and service industry worker, and office worker were independently associated with proactive practice (all *P* < 0.05). Those characteristics could help design future KAP interventions on vitD deficiency.

**Conclusions:**

This study demonstrated poor knowledge, positive attitude, and proactive practice regarding pediatric VitD deficiency among parents. Targeted interventions and educational programs should be developed to improve parental knowledge.

## 1 Introduction

The main source of vitamin D (VitD) in the human body is the conversion of 7-dehydrocholesterol by ultraviolet radiation from sunlight (1–3). The VitD deficiency rate in children and adolescents in China is 19.6%–78.1% (4, 5). In addition to its important relationship with calcium and phosphorus metabolism and bone health, VitD also plays an important role in immune regulation, protection of central nervous system function, and prevention of cardiovascular diseases, metabolic diseases, and tumors (1–3). For children, the potential complications of vitD deficiency also include rickets, bone mass loss, hyperparathyroidism, and cardiomyopathy (1, 2). Most school-age children have heavy schoolwork and a short time for outdoor activities, which might result in insufficient VitD synthesis (6). Since promoting adequate sun exposure and supplementing exogenous VitD are relatively simple means to avoid VitD deficiency, it is necessary to popularize relevant views and appeal to parents to pay attention to their children's adequate VitD supply (7). Previous studies on the KAP toward VitD were performed among adults (8–13); only one previous study reported that general practitioners had positive attitudes toward vitD supplementation in infants (14). Furthermore, as highlighted by a meta-analysis on the VitD status in China, there is a lack of clear and detailed guidelines on VitD in China, especially regarding children/adolescents (4). Determining the parental KAP regarding VtiD deficiency could contribute to determining more precise guidelines.

Therefore, this study aimed to investigate the KAP towards pediatric VitD deficiency among parents in Chengdu, China, and explore the risk factors of their knowledge, attitude, and practice.

## 2 Methods

### 2.1 Study design and participants

This cross-sectional survey was conducted at Chengdu Children's Specialized Hospital between November 2022 and January 2023. The inclusion criteria were (1) ≥18 years old and (2) parents of children under 14 years old. Parents with communication impairments or psychiatric disorders were excluded because of the difficulties in administering the questionnaire, possible doubts regarding whether consent to participate was actually informed, possible interference of the disorders with their perceptions, and possible interference of medication with such perception. Written informed consent was obtained from the parents before the survey. The parents were approached during routine health check-ups or hospitalization of their children. The study was approved by the Ethics Committee of Chengdu Children's Specialized Hospital (202206).

Chengdu Children's Specialized Hospital is a second-class hospital in Chengdu, which is responsible for the routine medical treatment of children in Chengdu, children's routine health care, and other routine diagnosis and treatment. Children with special needs or specific diseases (e.g., cancer, genetic diseases, congenital abnormalities, etc.) are referred to specialized tertiary hospitals. The average annual number of visits is more than 300,000 (most of them are common diseases, frequently-occurring diseases, especially respiratory infections), of which the average annual number of visits to the child health department is more than 8,000. Among them, the child health section undertakes the child health work of the community health service center. Data collection is performed by each department, including outpatient departments. The data sample is not much different from the data sample of the community service center.

### 2.2 Questionnaire

The questionnaire was designed with reference to relevant literature studies, guidelines, and expert consensus (15–17). The questionnaire was reviewed and modified by two experts (a pediatrician and a pediatric gastroenterologist). The questionnaire was pre-tested (*n* = 41) and showed a Cronbach’α of 0.90, indicating good internal consistency.

The final questionnaire was in Chinese and included 40 items. There were 15 items about demographic characteristics, 13 items on knowledge, nine items on attitude sections, and 10 items on practice. For knowledge items, 1 point was scored for each correct answer and 0 points for a wrong or unclear answer, with a possible score range of 0–13 points. For attitude items, a five-point Likert scale was used, and each item was scored from 5 points to 1 point according to the positive degree, with a possible score range of 9–45 points. For practice items, practice item #10 (P10) was to investigate “the ways to learn about VitD deficiency” and presented by descriptive analysis. The other nine items were scored from 5 points to 1 point, with a possible score range of 9–45 points. Knowledge, attitude, and practice scores ≥ 60% of the theoretical total scores were considered sufficient knowledge, positive attitude, and proactive practice.

### 2.3 Quality control and distribution process

The questionnaire was uploaded to the SoJump platform, and a link to the electronic questionnaire was subsequently output. The electronic questionnaires were disseminated in outpatient and inpatient departments, as well as on social platforms such as WeChat. If questions were encountered during answering, participants could ask the team members for help. The team members would provide interpretation to participants in time. After the data collection, the questionnaire was quality checked by the team members. Questionnaires with obvious logical errors, such as the age of 5 years old or a pattern of choosing exactly the same options, were considered invalid.

### 2.4 Statistical analysis

Stata 17.0 (Stata Corporation, College Station, TX, USA) was used for analysis. The continuous variables were presented as mean ± standard deviation (SD) and were analyzed by Student's *t*-test or one-way ANOVA if meeting a normal distribution or by Wilcoxon-Mann-Whitney test or Kruskal-Wallis analysis of variance if skewed distributed. The categorical variables were presented as *n* (percentage) and analyzed using the chi-square test. Variables with *P* < 0.05 in univariable logistic regression were included in the multivariable logistic regression. Multivariate logistic regression was conducted to determine the factors associated with knowledge, attitude, and practice. Subgroup analyses were performed among parents whose children were diagnosed with VitD deficiency. Two-sided *P* < 0.05 were considered statistically significant.

## 3 Results

A total of 624 questionnaires were collected, and 3 of them were excluded due to logical errors, resulting in 621 valid questionnaires (99.52%). Most parents were female (54.13%), 30–35 years old (56.04%), Han ethnicity (97.91%), living in urban areas (67.15%), married (84.22%), and with a bachelor's degree (46.05%). Among the parents, 308 (49.60%) reported that their children had a diagnosis of VitD deficiency, and 317 (51.05%) reported their children had undergone serum vitamin D testing in the past year (Table 1).

**Table 1:** Characteristics of the participants.

Characteristics	*n* (%) or mean ± SD	Knowledge score		Attitude score		Practice score	
		Mean ± SD	*P*	Mean ± SD	*P*	Mean ± SD	*P*
Total	621	6.13 ± 3.07		31.13 ± 6.20		27.47 ± 4.21	
Sex			0.003		0.033		<0.001
Male	260 (41.87)	5.72 ± 3.01		30.44 ± 6.47		26.80 ± 4.35	
Female	361 (58.13)	6.42 ± 3.07		31.63 ± 5.95		27.96 ± 4.03	
Age	33.99 ± 4.10		0.658		0.293		0.695
<30	75 (12.08)	6.13 ± 3.13		31.33 ± 6.00		27.12 ± 4.04	
[30, 35]	348 (56.04)	6.03 ± 3.01		30.77 ± 6.36		27.42 ± 4.39	
>35	198 (31.88)	6.28 ± 3.15		31.70 ± 5.97		27.70 ± 3.93	
Ethnicity			0.664		<0.001		0.017
Han	608 (97.91)	6.12 ± 3.07		31.35 ± 5.91		27.57 ± 4.09	
Other	13 (2.09)	6.31 ± 3.04		21.15 ± 10.30		23.08 ± 6.76	
Residence			<0.001		0.010		<0.001
Non-urban	204 (32.85)	5.06 ± 3.16		30.13 ± 6.51		26.49 ± 4.57	
Urban	417 (67.15)	6.65 ± 2.88		31.63 ± 5.99		27.95 ± 3.93	
Marital status			<0.001		<0.001		0.003
Married	523 (84.22)	6.48 ± 2.94		32.06 ± 5.36		27.86 ± 3.68	
Divorced/widowed	98 (15.78)	4.22 ± 3.03		26.20 ± 7.85		25.43 ± 5.94	
Education			<0.001		0.195		<0.001
Middle school and below	68 (10.95)	4.40 ± 2.97		29.46 ± 7.42		25.29 ± 5.36	
High school/technical secondary school	73 (11.76)	4.38 ± 3.55		30.08 ± 7.40		26.62 ± 5.58	
Junior college	113 (18.20)	5.81 ± 3.18		31.76 ± 6.08		26.50 ± 3.68	
Bachelor’s degree	286 (46.05)	7.08 ± 2.44		31.74 ± 5.17		28.62 ± 3.26	
Master’s degree and above	81 (13.04)	6.21 ± 3.15		30.46 ± 7.04		27.40 ± 4.18	
Spouse’s education			<0.001		0.445		0.002
Middle school and below	95 (15.30)	4.58 ± 3.01		29.99 ± 6.56		26.17 ± 4.80	
High school/technical secondary school	75 (12.08)	5.03 ± 3.58		30.43 ± 7.79		26.51 ± 5.22	
Junior college	121 (19.48)	6.36 ± 2.76		31.33 ± 6.05		27.62 ± 3.83	
Bachelor’s degree	210 (33.82)	7.04 ± 2.75		31.66 ± 5.75		28.18 ± 3.41	
Master’s degree and above	120 (19.32)	6.20 ± 2.95		31.36 ± 5.62		27.73 ± 4.34	
Job			<0.001		<0.001		<0.001
Professional and technical staff	96 (15.46)	7.90 ± 2.70		33.40 ± 5.17		29.45 ± 3.11	
Office worker	201 (32.37)	5.91 ± 2.95		31.74 ± 5.42		27.27 ± 3.90	
Commercial and service industry personnel	139 (22.38)	5.68 ± 3.04		30.10 ± 6.28		27.78 ± 3.96	
Other	185 (29.79)	5.77 ± 3.09		30.08 ± 7.01		26.44 ± 4.80	
Monthly per capita household income			0.003		0.784		0.249
<5,000	43 (6.92)	7.19 ± 3.47		30.81 ± 8.97		27.91 ± 4.29	
5,000–9,999	300 (48.31)	6.28 ± 2.81		31.47 ± 5.21		27.79 ± 3.85	
0,000–19,999	246 (39.61)	5.96 ± 3.06		31.05 ± 6.00		27.07 ± 4.44	
>20,000	32 (5.15)	4.56 ± 4.13		29.03 ± 10.38		27.09 ± 5.24	
Number of children			0.008		<0.001		0.088
1	333 (53.62)	6.41 ± 3.13		32.16 ± 6.27		27.72 ± 4.12	
2 and above	288 (46.38)	5.80 ± 2.97		29.95 ± 5.91		27.18 ± 4.29	
Age of the youngest child			0.260		0.116		0.101
Infancy (≤3 years old)	408 (65.70)	6.03 ± 2.94		31.23 ± 6.13		27.26 ± 4.23	
Preschool (3–6 years old)	115 (18.52)	6.11 ± 3.10		30.42 ± 5.66		27.75 ± 4.05	
School-age and above (6 years old and above)	98 (15.78)	6.52 ± 3.50		31.56 ± 7.02		28.03 ± 4.25	
Diagnosis status of VitD deficiency			<0.001		<0.001		0.001
Previously diagnosed and cured of VitD deficiency	226 (36.39)	6.39 ± 2.61		31.05 ± 5.77		27.84 ± 3.77	
Currently have VitD deficiency	82 (13.20)	4.90 ± 3.20		29.07 ± 7.15		25.71 ± 5.14	
Not previously diagnosed	251 (40.42)	6.63 ± 3.16		32.48 ± 5.53		27.98 ± 3.69	
Unclear	62 (9.98)	4.73 ± 3.25		28.73 ± 7.44		26.42 ± 5.44	
Has your child been tested for VitD in the last year			0.020		<0.001		0.001
Yes	317 (51.05)	6.17 ± 2.86		31.01 ± 5.39		27.91 ± 3.43	
VitD levels in children tested			0.181		0.488		0.123
Deficient [0, 12]	2 (0.99)	10.00 ± 2.83		35.50 ± 6.36		28.50 ± 4.95	
Insufficient [12, 20]	2 (0.99)	8.00 ± 1.41		34.50 ± 7.78		32.00 ± 0.00	
Sufficient [20, 100]	198 (98.02)	6.65 ± 2.61		30.97 ± 5.19		27.86 ± 3.32	
No	241 (38.81)	6.26 ± 3.34		32.24 ± 5.94		27.56 ± 4.42	
Unclear	63 (10.14)	5.38 ± 2.92		27.52 ± 9.02		24.97 ± 5.83	
Medical insurance type (multiple choice)			–		–		–
New cooperative medical insurance	138	5.74 ± 3.14		30.78 ± 6.24		26.57 ± 4.64	
Basic medical insurance for urban employees	474	6.23 ± 3.04		31.18 ± 6.22		27.70 ± 4.05	
Commercial insurance	103	7.24 ± 2.96		33.46 ± 6.16		28.41 ± 4.02	
No insurance	8	5.38 ± 3.07		28.88 ± 9.45		28.63 ± 3.78	
Other medical insurance types	10	4.90 ± 4.36		32.90 ± 5.11		26.40 ± 5.50	

**Figure 1:**
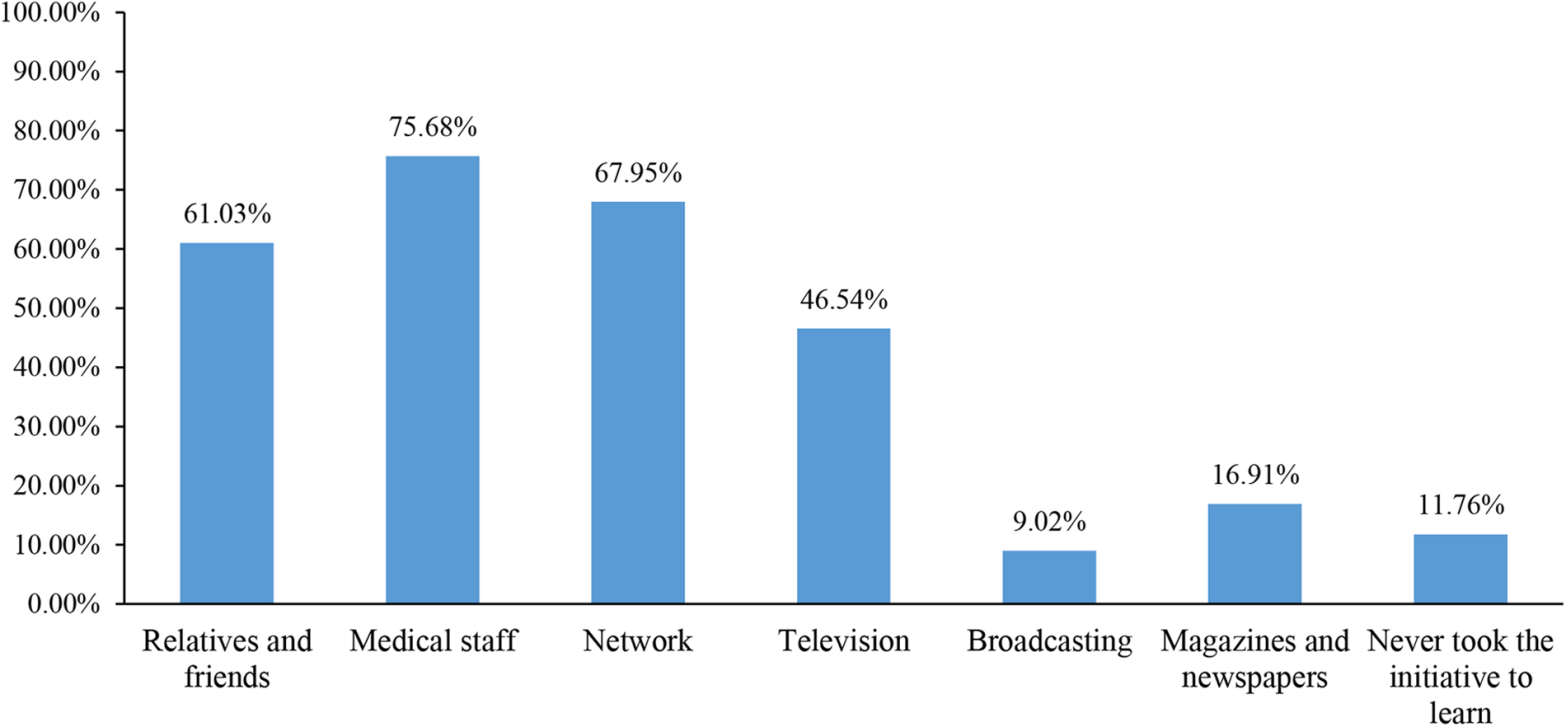
Access to learn about vitD deficiency (multiple choices).

The knowledge score was 6.13 ± 3.07 (47.15%, theoretical score range: 0–13), indicating poor knowledge. Higher knowledge scores were observed in females (*P* = 0.003), urban areas (*P* < 0.001), married (*P* < 0.001), and parents with one child (*P* = 0.008). There were two knowledge items with a correct rate lower than 20%: K7 (15.14%; “Winter and spring, high altitude and high latitude are risk factors for VitD deficiency, and additional VitD supplementation is required”), K9 (18.20%; “Drinking more milk can prevent VitD deficiency”) (Supplementary Table S1). The attitude score was 31.13 ± 6.20 (69.18%, theoretical score range: 9–45). Higher attitude scores were observed in females (*P* = 0.033), Han ethnicity (*P* < 0.001), urban areas (*P* = 0.010), married (*P* < 0.001), and parents with one child (*P* < 0.001). Most parents agreed that it is very important for children to maintain normal VitD levels (518, 83.41%) (Supplementary Table S2). The practice score was 27.47 ± 4.21 (61.04%, theoretical score range: 9–45). Higher practice scores were observed in females (*P* < 0.001), Han ethnicity (*P* = 0.017), urban areas (*P *< 0.001), and married (*P* = 0.003). More than half of the parents reported that they would follow the doctor's instructions to recheck and take VitD supplements if the doctor recommended (395, 63.61%) (Supplementary Table S3). The parents were mainly learning about VitD deficiency from medical staff and networks (Figure 1).

In the results of the subgroup analysis, the knowledge scores of parents whose children were diagnosed with VitD deficiency were 5.99 ± 2.83 (theoretical score range: 0–13, 46.08%) (Supplementary Table S4). There were five knowledge items with a correct rate lower than 20%: K4 (17.53%; “VitD supplements at least 400U every day are needed for children before the age of three, and no longer after the age of three”), K5 (17.53%; “Oral VitD supplements are only needed after children are diagnosed with VitD deficiency”), K7 (13.96%; “Winter and spring, high altitude and high latitude are risk factors for VitD deficiency, and additional VitD supplementation is required”), K9 (14.29%; “Drinking more milk can prevent VitD deficiency”), and K10 (19.48%; “VitD supplementation alone can prevent rickets without calcium supplementation”). Most parents agreed that it is very important for children to maintain normal VitD levels (253, 82.14) (Supplementary Table S5). More than half of the parents reported that they would follow the doctor's instructions to recheck and take VitD supplements if the doctor recommended (183, 59.42%) (Supplementary Table S6).

**Figure 2:**
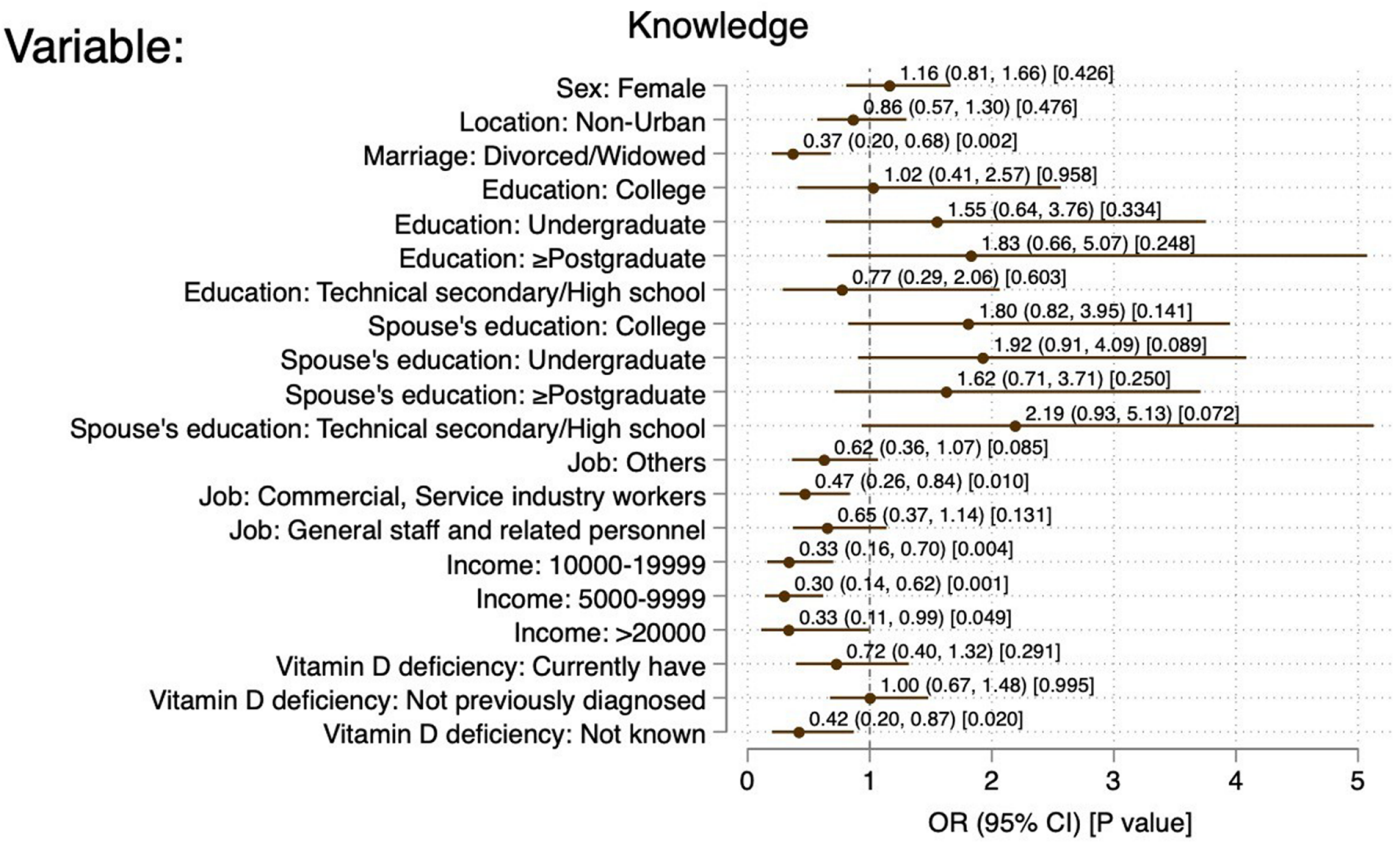
Forest plot of factors associated with good knowledge. References: Sex: male; location: urban; marriage: married; education: primary school and below; spouse's education: primary school and below; job: professional and technical staff; income: 10,000-19,999; VitD deficiency: previously diagnosed and cured of VitD deficiency.

**Figure 3:**
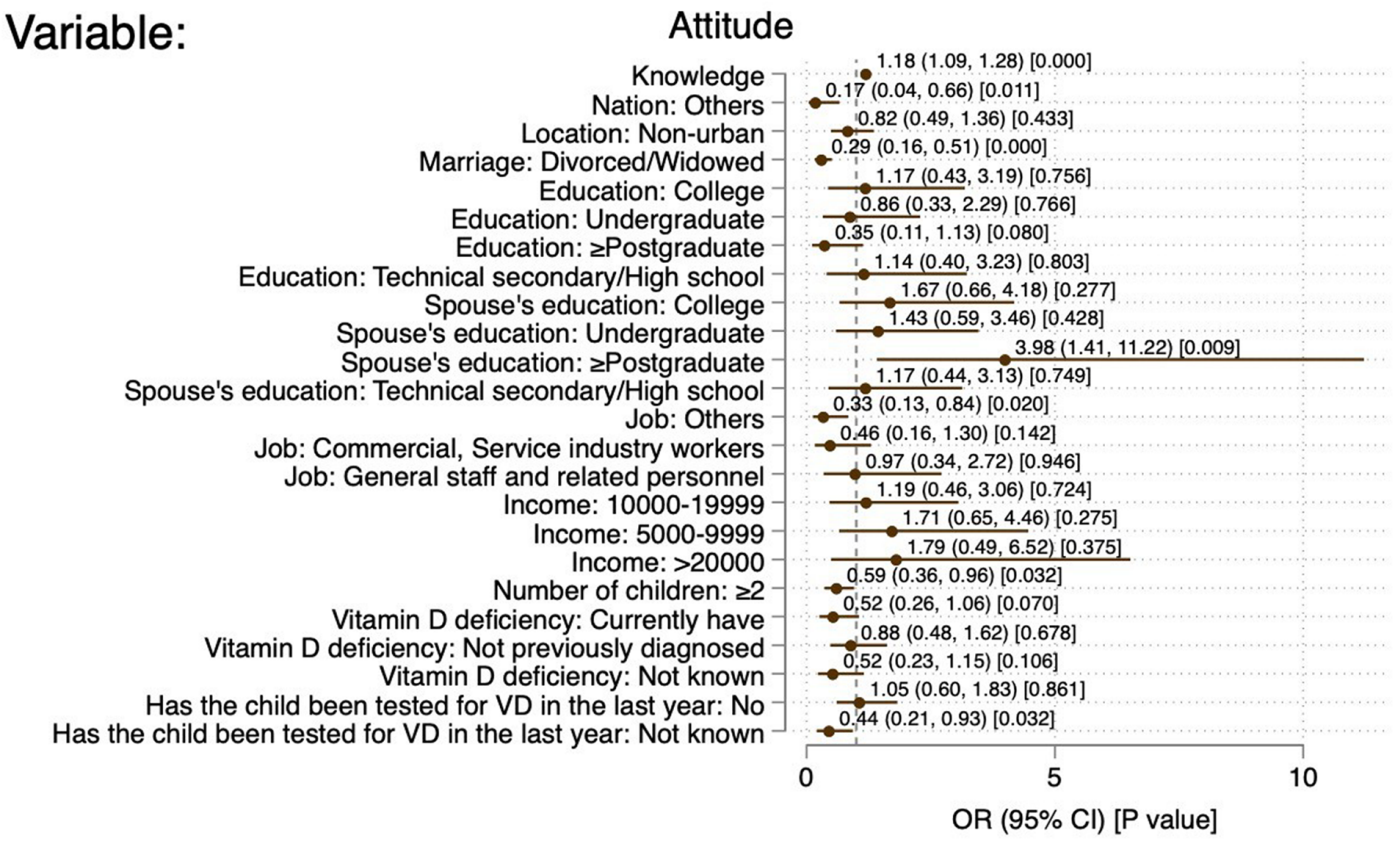
Forest plot of factors associated with positive attitude. References: location: urban; marriage: married; education: primary school and below; spouse's education: primary school and below; job: professional and technical staff; income: 10,000-19,999; number of children: 1; VitD deficiency: previously diagnosed and cured of VitD deficiency; has the child been tested for VD in the past years: yes.

**Figure 4:**
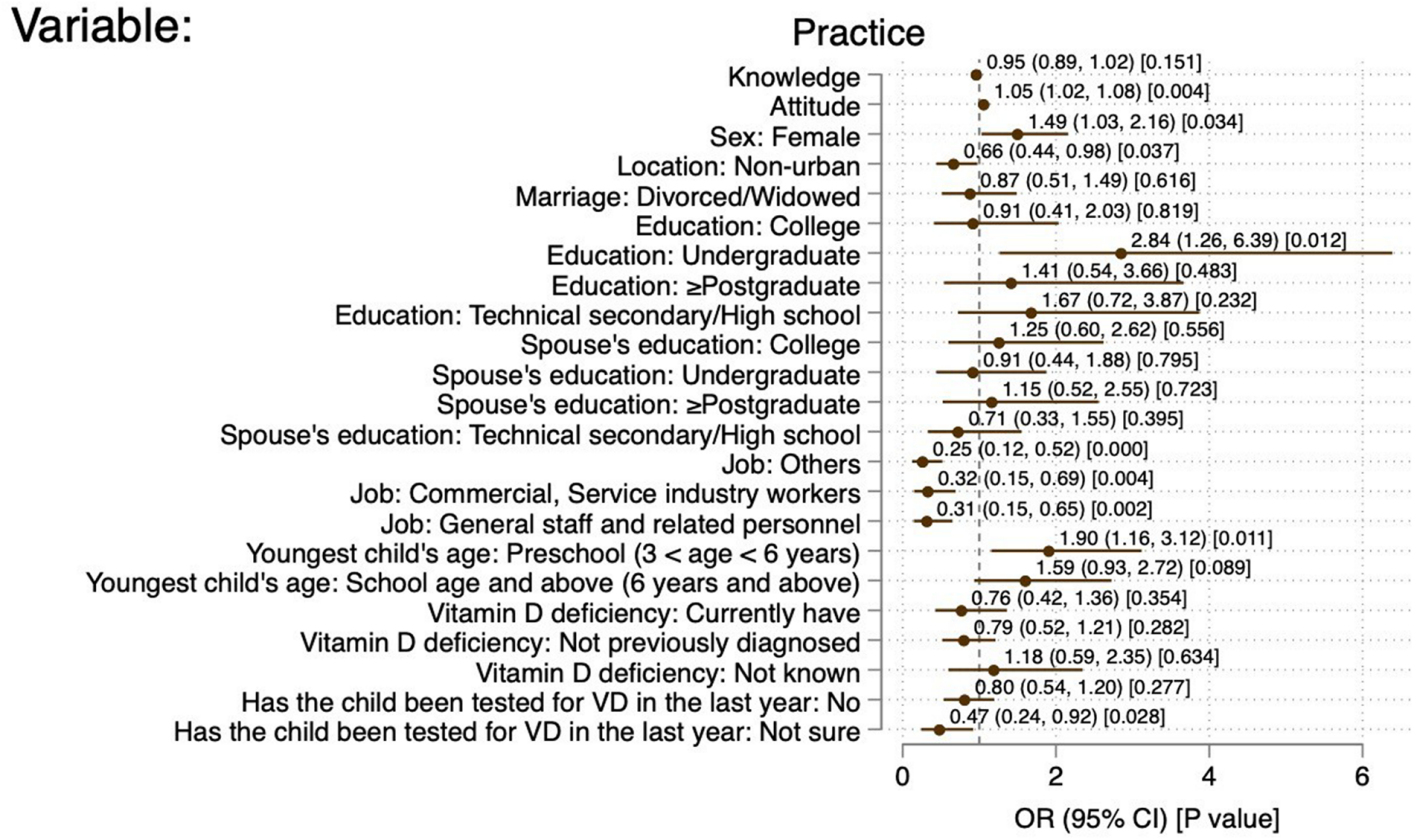
Forest plot of factors associated with positive practice. References: sex: male; location: urban; marriage: married; education: primary school and below; spouse's education: primary school and below; job: professional and technical staff; youngest child's age: infancy (≤3 years); VitD deficiency: previously diagnosed and cured of VitD deficiency; has the child been tested for VD in the past years: yes.

Commercial and service industry workers (OR = 0.47, 95% CI: 0.26–0.48, *P* = 0.010), monthly income of 5,000–9,999 CNY (OR = 0.30, 95% CI: 0.14–0.62, *P* < 0.01), 10,000–19,999 CNY (OR = 0.33, 95% CI: 0.16–0.70, *P* = 0.001), and ≥20,000 CNY (OR = 0.33, 95% CI: 0.11–0.99, *P* = 0.049) were independently associated with sufficient knowledge. The knowledge scores (OR = 1.18, 95% CI: 1.09–1.28, *P* < 0.001), ethnic minorities (OR = 0.17, 95% CI: 0.04–0.66, *P* = 0.011), divorces/widowed (OR = 0.29, 95% CI: 0.16–0.51, *P* < 0.001), and spouse with master degree or above (OR = 3.98, 95% CI: 1.41–11.22, *P* = 0.009) were independently associated with positive attitude. The attitude scores (OR = 1.05, 95% CI: 1.02–1.08, *P* = 0.004), female (OR = 1.49, 95%CI: 1.03–2.16, *P* = 0.034), non-urban (OR = 0.66, 95% CI: 0.44–0.98, *P* = 0.037), undergraduate education (OR = 2.84, 95% CI: 1.26–6.39, *P* = 0.012), commercial and service industry worker (OR = 0.32, 95% CI: 0.15–0.69, *P* = 0.004), and office worker (OR = 0.31, 95% CI: 0.15–0.65, *P* = 0.002) were independently associated with proactive practice (Figures 2–4).

## 4 Discussion

This study demonstrated the poor knowledge, positive attitudes, and proactive practice regarding pediatric VitD deficiency among parents. Parents whose children were diagnosed with vitD deficiency also had poor knowledge regarding VitD deficiency. The results of the study may help healthcare providers design effective interventions to improve the KAP of parents toward pediatric vitamin D deficiency.

Reaching the target VitD intake is important for children to avoid developmental issues and complications later in life (1, 2). China has specific guidelines regarding VitD deficiency (15–17), but VitD deficiency remains a public health issue, which might be due to improper parental implementation of prevention (18, 19). Appropriate knowledge of the risks associated with VitD deficiency is paramount to implementing VitD deficiency prevention. A previous study showed that most women in Saudi Arabia show poor knowledge and unfavorable attitudes toward VitD deficiency (20). There were no previous studies in China specifically on the KAP towards VitD deficiency, but a previous study showed that mothers’ KAP on nutrition, in general, was associated with the 25(OH)D levels and the incidence of rickets in the children in China (21). In line with these previous studies, this study also showed poor parental knowledge of VitD deficiency (even in those with children diagnosed with VtD deficiency). Regarding attitudes and practices, Cicek et al. (22) reported poor attitudes and practices among mothers in Konya (Turkey). Elsobkey et al. (23) reported poor attitudes and practices regarding VitD deficiency among mothers of children with cerebral palsy in Egypt, but they also reported that an education intervention could improve their attitudes and practices. The present study contradicted these two studies (22, 23) and showed favorable attitudes and proactive practice, indicating that even without proper knowledge, the parents appear to follow the advice they hear from different sources such as healthcare providers, social networks, and advertising.

Previous studies in Jordan and Egypt showed that maternal education was associated with knowledge about VitD deficiency (24, 25), which was observed in the univariable analysis of this study but not in the multivariable analysis. In the present study, higher KAP was generally associated with divorced or widowed but also with working and higher incomes, probably because of less time the parents can spend learning about VitD deficiency. The inverse relationship between income and VitD deficiency knowledge could also be related to better access to better healthcare. Another reason might also be that families with a higher income have a higher chance of better nutrition and adequate VitD intake; thus, being less exposed to the risk of VitD deficiency, such people would have lower knowledge of the condition. Higher education was associated with higher attitude and practice scores. Having had the child tested in the past year for VitD deficiency was associated with lower attitude and practice scores, probably because of fewer worries about the condition. The present study also showed that medical staff, networks, relatives and friends, and television are the main sources of information for parents. Thus, the parents could be reached through these sources for KAP improvements, but other sources should also be used to reach as many parents as possible.

A study showed that an education program on VitD deficiency could improve the knowledge of the disease (25). The present study highlighted several knowledge areas that were deficient, including proper VitD supplementation, appropriate blood 25(OH)D levels, seasons at risk, rickets prevention, and the involvement of VitD in general health. Future teaching interventions should cover those areas. Especially parents of children diagnosed with VitD deficiency should receive proper education to help improve the condition of their children and avoid recurrence.

The possible confounders in this study are those usually related to health literacy, i.e., education, non-healthcare jobs, and socioeconomic status (26–28). They were taken into account in this study, which is a strength of this study. On the other hand, it is true that information on comorbidities, physical activity of the children, eating habits, and diet information is missing. This study had other limitations. It was performed at a single center. The education of the parents was high, which may limit the generalization of the findings. The present study did not collect data about the use of fortification and vitamin D-fortified foods by the parents of their children. This study was limited by the social desirability bias, like all KAP studies, in which the participants can be tempted to respond what they should respond instead of what they were actually doing (29, 30). However, the present study could serve as a pilot study to determine the impact of future interventions.

In conclusion, parents demonstrated poor knowledge, positive attitude, and active practice regarding VitD deficiency in children. This study identified specific areas in KAP that could be intervened in future teaching interventions. Interventions should improve the public health condition of the children in Chengdu, China.

## Data Availability

The original contributions presented in the study are included in the article/Supplementary Material, further inquiries can be directed to the corresponding author.
