# Supplementary material for: Knowledge, attitude, and practice toward pediatric vitamin D deficiency among parents
Source: Front Pediatr. 2024 Jun 28;12:1393488. doi: 10.3389/fped.2024.1393488 (PMC11240275; doi:10.3389/fped.2024.1393488)
Supplement: Supplementary file 1 [file Datasheet1.docx]

**Supplementary** Table 1. Knowledge of parents.

| **Knowledge** | **Correct, n (%)** |
| --- | --- |
| **Total** | 6.13 ± 3.07 |
| K1. Concept of VitD deficiency. | 442 (71.18) |
| K2. Children are prone to VitD deficiency. | 405 (65.22) |
| K3. Severe rickets can leave varying degrees of skeletal deformities. | 403 (64.90) |
| K4. VitD supplements at least 400U every day are needed for children before the age of three, and no longer after the age of three. | 143 (23.03) |
| K5. Oral VitD supplements are only needed after children are diagnosed with VitD deficiency. * | 171 (27.54) |
| K6. Serum 25-hydroxy VitD levels in children are appropriate at 50 to 125 nmol/L. | 345 (55.56) |
| K7. Winter and spring, high altitude, and high latitude are risk factors for VitD deficiency, and additional VitD supplementation is required. * | 94 (15.14) |
| K8. Sunlight exposure is the main method for the body to obtain VitD. When sunlight exposure is limited, additional VitD supplementation is appropriate. | 400 (64.41) |
| K9. Drinking more milk can prevent VitD deficiency. * | 113 (18.20) |
| K10. VitD supplementation alone can prevent rickets without calcium supplementation* | 150 (24.15) |
| K11. VitD deficiency is related to the occurrence of respiratory tract infection and intestinal infections. | 359 (57.81) |
| K12. Effect of VitD. | 380 (61.19) |
| K13. Symptoms of VitD deficiency. | 399 (64.25) |

* The item was reversely scored.

**Supplementary** Table 2. Attitude of parents.

| Items | Strongly agree, n (%) | Agree, n (%) | Neutral, n (%) | Disagree, n (%) | Strongly disagree, n (%) |
| --- | --- | --- | --- | --- | --- |
| A1. It is very important for children to maintain normal VitD levels. | 223 (35.91) | 295 (47.50) | 61 (9.82) | 33 (5.31) | 9 (1.45) |
| A2. You are worried that your child lacks VitD. | 195 (31.40) | 224 (36.07) | 117 (18.84) | 54 (8.70) | 31 (4.99) |
| A3. You pay attention to your child’s VitD status. | 197 (31.72) | 225 (36.23) | 113 (18.20) | 52 (8.37) | 34 (5.48) |
| A4. To prevent VitD deficiency, it is necessary to take more VitD-rich foods. | 214 (34.46) | 212 (34.14) | 102 (16.43) | 56 (9.02) | 37 (5.96) |
| A5. Daily outdoor exercise is necessary to prevent VitD deficiency. | 212 (34.14) | 231 (37.20) | 94 (15.14) | 53 (8.53) | 31 (4.99) |
| A6. It is not necessary for children to take oral VitD supplements on a daily basis. | 25 (4.03) | 43 (6.92) | 99 (15.94) | 273 (43.96) | 181 (29.15) |
| A7. It is important to treat VitD deficiency promptly. | 221 (35.59) | 234 (37.68) | 88 (14.17) | 45 (7.25) | 33 (5.31) |
| A8. You trust the doctor’s advice on VitD supplementation. | 227 (36.55) | 221 (35.59) | 104 (16.75) | 37 (5.96) | 32 (5.15) |
| A9. You want to learn more about VitD deficiency and how to take VitD supplements scientifically. | 231 (37.20) | 213 (34.30) | 93 (14.98) | 47 (7.57) | 37 (5.96) |

**Supplementary** Table 3. Practice of parents.

| Items | Always / Totally conforming, n (%) | Often / Partially Conforming, n (%) | Sometimes / Neutral, n (%) | Hardly / Partially not conforming, n (%) | Unclear / Totally not conforming, n (%) |
| --- | --- | --- | --- | --- | --- |
| P1. You (or your spouse) took the initiative to supplement VitD during pregnancy. | 115 (18.52) | 283 (45.57) | 157 (25.28) | 51 (8.21) | 15 (2.42) |
| P2. The time of your child’s daily outdoor activities in the daytime is: | 38 (6.12) | 190 (30.60) | 300 (48.31) | 69 (11.11) | 24 (3.86) |
| P3. The frequency of your child taking VitD supplements is: | 149 (23.99) | 211 (33.98) | 153 (24.64) | 79 (12.72) | 29 (4.67) |
| P4. The frequency of your child eating deep-sea fish every week is: | 48 (7.73) | 136 (21.90) | 355 (57.17) | 71 (11.43) | 11 (1.77) |
| P5. The frequency of your child eating eggs every week is: | 210 (33.82) | 312 (50.24) | 76 (12.24) | 16 (2.58) | 7 (1.13) |
| P6. You will pay attention to the children’s nutrition to supplement VitD, such as eating more deep-sea fish, eggs, etc. | 143 (23.03) | 224 (36.07) | 154 (24.80) | 61 (9.82) | 39 (6.28) |
| P7. You often take the initiative to learn about VitD supplementation. | 118 (19.00) | 223 (35.91) | 166 (26.73) | 69 (11.11) | 45 (7.25) |
| P8. If symptoms of VitD deficiency appear, you will take the initiative to bring your child to seek medical attention. | 183 (29.47) | 197 (31.72) | 162 (26.09) | 53 (8.53) | 26 (4.19) |
| P9. If your doctor recommends it, you will follow the doctor’s instructions to recheck and take VitD supplements. | 176 (28.34) | 219 (35.27) | 133 (21.42) | 65 (10.47) | 28 (4.51) |

**Supplementary Table 4.** Subgroup analysis: knowledge scores of parents whose children were diagnosed VitD deficiency.

| Knowledge | Correct, n (%) |
| --- | --- |
| Total | 5.99 ± 2.85 |
| K1. Concept of VitD deficiency. | 220 (71.43) |
| K2. Children are prone to VitD deficiency. | 200 (64.94) |
| K3. Severe rickets can leave varying degrees of skeletal deformities. | 199 (64.61) |
| K4. VitD supplements at least 400U every day are needed for children before the age of three, and no longer after the age of three. | 54 (17.53) |
| K5. Oral VitD supplements are only needed after children are diagnosed with VitD deficiency. * | 54 (17.53) |
| K6. Serum 25-hydroxy VitD levels in children are appropriate at 50 to 125 nmol/L. | 193 (62.66) |
| K7. Winter and spring, high altitude, and high latitude are risk factors for VitD deficiency, and additional VitD supplementation is required. * | 43 (13.96) |
| K8. Sunlight exposure is the main method for the body to obtain VitD. When sunlight exposure is limited, additional VitD supplementation is appropriate. | 199 (64.61) |
| K9. Drinking more milk can prevent VitD deficiency. * | 44 (14.29) |
| K10. VitD supplementation alone can prevent rickets without calcium supplementation* | 60 (19.48) |
| K11. VitD deficiency is related to the occurrence of respiratory tract infections and intestinal infections. | 187 (60.71) |
| K12. Effect of VitD. | 200 (64.94) |
| K13. Symptoms of VitD deficiency. | 193 (62.66) |

**Supplementary Table 5.** Subgroup analysis: attitude of parents whose children were diagnosed VitD deficiency.

| Items | Strongly agree, n (%) | Agree, n (%) | Neutral, n (%) | Disagree, n (%) | Strongly disagree, n (%) |
| --- | --- | --- | --- | --- | --- |
| A1. It is very important for children to maintain normal VitD levels. | 98 (31.82) | 155 (50.32) | 34 (11.04) | 15 (4.87) | 6 (1.95) |
| A2. You are worried that your child lacks VitD. | 94 (30.52) | 112 (36.36) | 58 (18.83) | 26 (8.44) | 18 (5.84) |
| A3. You pay attention to your child’s VitD status. | 102 (33.12) | 109 (35.39) | 55 (17.86) | 24 (7.79) | 18 (5.84) |
| A4. To prevent VitD deficiency, it is necessary to take more VitD-rich foods. | 95 (30.84) | 101 (32.79) | 53 (17.21) | 34 (11.04) | 25 (8.12) |
| A5. Daily outdoor exercise is necessary to prevent VitD deficiency. | 88 (28.57) | 116 (37.66) | 55 (17.86) | 33 (10.71) | 16 (5.19) |
| A6. It is not necessary for children to take oral VitD supplements on a daily basis. | 14 (4.55) | 19 (6.17) | 50 (16.23) | 128 (41.56) | 97 (31.49) |
| A7. It is important to treat VitD deficiency promptly. | 92 (29.87) | 121 (39.29) | 55 (17.86) | 21 (6.82) | 19 (6.17) |
| A8. You trust the doctor’s advice on VitD supplementation. | 107 (34.74) | 99 (32.14) | 67 (21.75) | 18 (5.84) | 17 (5.52) |
| A9. You want to learn more about VitD deficiency and how to take VitD supplements scientifically. | 109 (35.39) | 104 (33.77) | 49 (15.91) | 23 (7.47) | 23 (7.47) |

**Supplementary Table 6.** Subgroup analysis: practice of parents whose children were diagnosed VitD deficiency.

| Items | Always / Totally conforming, n (%) | Often / Partially Conforming, n (%) | Sometimes / Neutral, n (%) | Hardly / Partially not conforming, n (%) | Unclear / Totally not conforming, n (%) |
| --- | --- | --- | --- | --- | --- |
| P1. You (or your spouse) took the initiative to supplement VitD during pregnancy. | 50 (16.23) | 155 (50.32) | 78 (25.32) | 16 (5.19) | 9 (2.92) |
| P2. The time of your child’s daily outdoor activities in the daytime is: | 17 (5.52) | 100 (32.47) | 153 (49.68) | 24 (7.79) | 14 (4.55) |
| P3. The frequency of your child taking VitD supplements is: | 63 (20.45) | 116 (37.66) | 77 (25.00) | 43 (13.96) | 9 (2.92) |
| P4. The frequency of your child eating deep-sea fish every week is: | 29 (9.42) | 73 (23.70) | 177 (57.47) | 26 (8.44) | 3 (0.97) |
| P5. The frequency of your child eating eggs every week is: | 106 (34.42) | 158 (51.30) | 36 (11.69) | 5 (1.62) | 3 (0.97) |
| P6. You will pay attention to the children’s nutrition to supplement VitD, such as eating more deep-sea fish, eggs, etc. | 73 (23.70) | 108 (35.06) | 83 (26.95) | 20 (6.49) | 24 (7.79) |
| P7. You often take the initiative to learn about VitD supplementation. | 61 (19.81) | 111 (36.04) | 83 (26.95) | 25 (8.12) | 28 (9.09) |
| P8. If symptoms of VitD deficiency appear, you will take the initiative to bring your child to seek medical attention. | 81 (26.30) | 98 (31.82) | 84 (27.27) | 25 (8.12) | 20 (6.49) |
| P9. If your doctor recommends it, you will follow the doctor’s instructions to recheck and take VitD supplements. | 78 (25.32) | 105 (34.09) | 74 (24.03) | 36 (11.69) | 15 (4.87) |
